# Invasive Assessment of Right Ventricular to Pulmonary Artery Coupling Improves 1-year Mortality Prediction After Transcatheter Aortic Valve Replacement and Anticipates the Persistence of Extra-Aortic Valve Cardiac Damage

**DOI:** 10.1016/j.shj.2024.100282

**Published:** 2024-03-15

**Authors:** Mark Lachmann, Amelie Hesse, Teresa Trenkwalder, Erion Xhepa, Tobias Rheude, Moritz von Scheidt, Héctor Alfonso Alvarez Covarrubias, Elena Rippen, Oksana Hramiak, Costanza Pellegrini, Tibor Schuster, Shinsuke Yuasa, Heribert Schunkert, Adnan Kastrati, Christian Kupatt, Karl-Ludwig Laugwitz, Michael Joner

**Affiliations:** a First Department of Medicine, Klinikum rechts der Isar, Technical University of Munich, Munich, Germany; b DZHK (German Centre for Cardiovascular Research), Partner Site Munich Heart Alliance, Munich, Germany; c Department of Cardiology, Deutsches Herzzentrum München, Technical University of Munich, Munich, Germany; d Specialized Department of Cardiology, Ternopil City Communal Hospital №2, Ternopil National Medical University, Ternopil, Ukraine; e Department of Family Medicine, McGill University, Montreal, Canada; f Department of Cardiology, Keio University School of Medicine, Tokyo, Japan

**Keywords:** Aortic stenosis, Right ventricular to pulmonary artery coupling, Transcatheter aortic valve replacement

## Abstract

**Background:**

The interplay between the right ventricle and the pulmonary artery, known as right ventricular to pulmonary artery (RV-PA) coupling, is crucial for assessing right ventricular systolic function against the afterload from the pulmonary circulation. Pulmonary artery pressure levels are ideally measured by right heart catheterization. Yet, echocardiography represents the most utilized method for evaluating pulmonary artery pressure levels, albeit with limitations in accuracy. This study therefore aims to evaluate the prognostic significance of right ventricular to pulmonary artery (RV-PA) coupling expressed as tricuspid annular plane systolic excursion (TAPSE) related to systolic pulmonary artery pressure (sPAP) levels measured by right heart catheterization (TAPSE/sPAP_invasive_) or estimated by transthoracic echocardiography (TAPSE/sPAP_echocardiography_) in patients with severe aortic stenosis undergoing transcatheter aortic valve replacement (TAVR).

**Methods:**

Using data from a bicentric registry, this study compares TAPSE/sPAP_invasive_ vs. TAPSE/sPAP_echocardiography_ in predicting 1-year all-cause mortality after TAVR.

**Results:**

Among 333 patients with complete echocardiography and right heart catheterization data obtained before TAVR, their mean age was 79.8 ± 6.74 years, 39.6% were female, and general 1-year survival was 89.8%. sPAP_invasive_ and sPAP_echocardiography_ showed only moderate correlation (Pearson correlation coefficient *R*: 0.53, *p* value: <0.0001). TAPSE/sPAP_invasive_ was superior to TAPSE/sPAP_echocardiography_ in predicting 1-year all-cause mortality after TAVR (area under the curve: 0.662 vs. 0.569, *p* value: 0.025). Patients with reduced TAPSE/sPAP_invasive_ levels (< 0.365 mm/mmHg) evidenced significantly lower 1-year survival rates than patients with preserved TAPSE/sPAP_invasive_ levels (81.8 vs. 93.6%, *p* value: 0.001; hazard ratio for 1-year mortality: 3.09 [95% confidence interval: 1.55-6.17]). Echocardiographic follow-up data revealed that patients with reduced RV-PA coupling suffer from persistent right ventricular dysfunction (TAPSE: 16.6 ± 4.05 mm vs. 21.6 ± 4.81 mm in patients with preserved RV-PA coupling) and severe tricuspid regurgitation (diagnosed in 19.7 vs. 6.58% in patients with preserved RV-PA coupling).

**Conclusions:**

RV-PA coupling expressed as TAPSE/sPAP_invasive_ can refine stratification of severe aortic stenosis patients into low-risk and high-risk cohorts for mortality after TAVR. Moreover, it can help to anticipate persistent extra-aortic valve cardiac damage, which will demand further treatment.

## Highlights

• RV-PA coupling is useful for mortality prediction in patients undergoing TAVR.
• Right-sided cardiac damage is likely to persist in patients with reduced RV-PA coupling.
• RV-PA coupling can guide the timing of intervention and the identification of further treatment targets.

## Graphical abstract

Prognostic significance of right ventricular to pulmonary artery coupling in patients with severe aortic stenosis undergoing TAVR (graphical summary). Stratification according to TAPSE/sPAP_invasive_ does not only improve prediction for 1-year mortality in patients with severe aortic stenosis undergoing TAVR, but it also helps to identify patients with persistent right-sided cardiac damage such as right ventricular systolic dysfunction and severe TR after TAVR. Stratification according to TAPSE/sPAP_invasive_ ratio could thus guide both timing of intervention (ideally before the fixation of irreversible cardiac damage) and identification of additional treatment targets (such as severe TR). Abbreviations: AVA, aortic valve area; AVG_mean_, mean aortic valve gradient; COPD, chronic obstructive pulmonary disease; LA area, left atrial area; LVEDD, left ventricular end-diastolic diameter; LVEF, left ventricular ejection fraction; mPAP, mean pulmonary artery pressure; PVR, pulmonary vascular resistance; RA area, right atrial area; sPAP, systolic pulmonary artery pressure; TAPSE, tricuspid annular plane systolic excursion.

**Figure:**
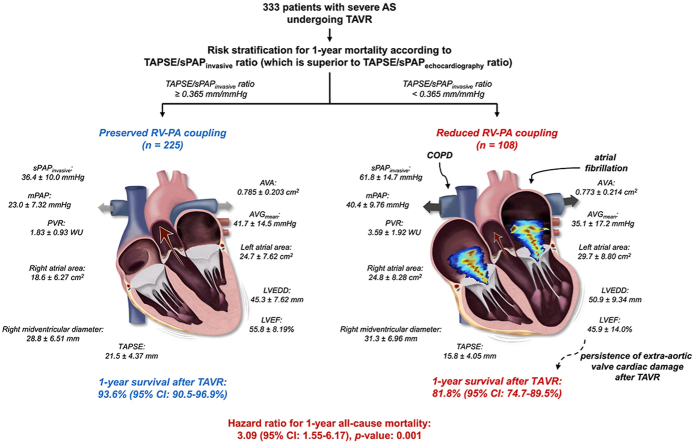

## Introduction

Calcific aortic stenosis (AS) is among the most common cardiovascular diseases in developed countries, following coronary artery disease and systemic arterial hypertension. Its prevalence increases with age, affecting up to 9.8% of individuals aged 80 years and older, reflecting its degenerative nature.^1^, ^2^, ^3^ In the context of the lack of strategies to slow down disease progression, the natural course of degenerative AS is typically characterized by a long latency period, during which the left ventricle adapts to progressive aortic valve narrowing with concentric muscle hypertrophy.^4^ In time, left ventricular remodeling becomes maladaptive and a cascade of extra-aortic valve cardiac damages is triggered: backward transmission of left-sided filling pressures results in pulmonary hypertension (PH) as found in 50% of patients with severe AS.^5^ Initially, the right ventricle reacts to PH with increased contractility in a form of homeometric adaption, but right ventricular compensation processes will eventually fail and lead to right heart dilatation with impairment of right ventricular function and tricuspid regurgitation (TR).^6^

Since transcatheter aortic valve replacement (TAVR) has been established as a safe treatment option in inoperable patients,^7^ a growing body of evidence suggests that the extent of extra-aortic valve cardiac damage determines prognosis.^8^^,^^9^ Phenotyping of patients with severe AS according to the extra-aortic valve cardiac damage — either by a traditional staging classification assuming a linear sequence of accumulated pathologies or by an unsupervised machine learning approach also incorporating the aggravating impact of, for example, comorbidities and genetic predisposition — further revealed that the cardiac damage is only partially reversible upon TAVR.^10^^,^^11^

Importantly, the pulmonary circulation and the right heart have long been regarded as separate units—practically overlooking that the relatively thin and more compliant right ventricle is highly sensitive to the imposed pressure load from the pulmonary circulation. The right ventricular to pulmonary artery (RV-PA) coupling concept aims to provide more physiology-based information by connecting the right ventricular systolic performance to a specific degree of pressure burden; thus, the RV-PA coupling concept promises to better reflect right ventricular contractability under the given circumstances of elevated pulmonary artery pressure levels with subsequent afterload and volume challenges to the right ventricle. Reduced RV-PA coupling indicates that the right ventricle is operating under unfavorable hemodynamic conditions, enduring high pressures despite impaired systolic function, a situation that often heralds a poor prognosis for affected patients. Expressed as the ratio of tricuspid annular plane systolic excursion (TAPSE) as a surrogate parameter for longitudinal right ventricular fiber shortening in relation to echocardiographic assessment of systolic pulmonary artery pressure (sPAP) levels, the prognostic significance of the resulting TAPSE/sPAP ratio has been shown in patients undergoing transcatheter intervention for various valvular heart diseases such as severe AS, mitral regurgitation (MR) and TR.^12^, ^13^, ^14^ However, echocardiography tends to underestimate sPAP levels in patients with severe TR, because a huge tricuspid valve regurgitant orifice area results in rapid pressure equalization between right ventricle and right atrium.^15^^,^^16^ Current guidelines therefore recommend right heart catheterization to measure pulmonary artery pressure levels and to diagnose PH.^17^ In patients with severe TR undergoing transcatheter intervention, it has already been shown that the prognostic value of the RV-PA coupling concept was improved if sPAP levels were measured invasively by right heart catheterization (instead of echocardiographic estimation).^18^

This study focusing on patients with severe AS undergoing TAVR aimed to explore 1) whether invasive assessment of sPAP levels by right heart catheterization improves prognostic resolution of RV-PA coupling regarding 1-year mortality after TAVR, and 2) whether stratification according to RV-PA coupling identifies patients with irreversible extra-aortic valve cardiac damage — possibly addressing the crucial question “How late is too late for a damaged heart to recover?”.

## Methods

### Study Population

This is a retrospective cohort study drawing on prospectively and systematically collected echocardiographic and hemodynamic data from patients with severe AS. Enrolled patients underwent TAVR for severe AS at 2 tertiary centers in Munich, Germany, between January 2014 and December 2020. Patients were included in the registry only after written informed consent was received. Since this study aimed to quantify the difference in predictive capacity of RV-PA coupling indices as assessed by echocardiography alone or by echocardiography in combination with right heart catheterization, only patients with both, that is complete preprocedural echocardiography and right heart catheterization, obtained before TAVR, were included in this study. Planned and conducted in conformity with the Declaration of Helsinki, this study was approved by the local ethics committee.

### Echocardiographic Analysis

All echocardiographic studies were conducted by experienced institutional cardiologists as part of routine clinical practice. A “classical” low-flow, low-gradient AS was defined as having an aortic valve area (AVA) ≤ 1.0 cm^2^, a mean aortic valve gradient < 40 mmHg, and a left ventricular ejection fraction <50%.^19^ This definition corresponds to stage D2 disease as per the American Heart Association/American College of Cardiology classification system.^20^ Echocardiographic sPAP levels were calculated by adding peak systolic pressure gradients between the right ventricle and right atrium (estimated from the continuous wave Doppler profile of the TR jet) to right atrial pressure levels. Right atrial pressure, in turn, was estimated by the diameter and collapsibility of the inferior vena cava. Right ventricular systolic function was assessed based on TAPSE measurements. Follow-up echocardiography was routinely performed 6 ​months after TAVR, unless specific interests (e.g. diagnostic evaluation of cardiac decompensation) justified an earlier or repeated investigation.

### Invasive PH Assessment

A 7 French Swan-Ganz catheter was routinely used for preprocedural right heart catheterization via femoral access. Systolic and diastolic pulmonary artery pressure (sPAP and dPAP) levels were directly recorded. Mean pulmonary artery pressure (mPAP) levels were calculated as mPAP = dPAP +1/3 x (sPAP–dPAP). Mean postcapillary wedge pressure was assessed over the entire cardiac cycle. Cardiac output was determined using the thermodilution technique or indirect Fick method, as appropriate.

### Assessment of Extra-Aortic Valve Cardiac Damage

To evaluate the severity of extra-aortic valve cardiac damage, we adapted the sequential staging classification from Généreux *et al*,^8^ categorizing patients into 4 stages of disease progression. Before assigning patients to these stages, missing data needed for the classification were imputed using a well-established random forest algorithm.^21^ For subsequent analyses, however, including baseline characteristic comparisons, only original, non-imputed data were used to ensure the integrity of our comparisons.

### Clinical Endpoint Definition

As an elderly patient population was studied, 1-year all-cause mortality following TAVR was defined as a clinically meaningful primary outcome measure. Survival data were regularly obtained from the German Civil Registry, or from general practitioners, hospitals, and practice cardiologists for patients from foreign countries.

### Statistical Analysis

Categorical variables are presented as numbers and/or frequencies (%), and continuous variables are given as means ± standard deviation and 95% confidence interval (CI).

Chi-square or Fisher exact test were used to evaluate the association between categorical variables, and independent-samples Wilcoxon test was used for comparison of continuous variables. Pairwise comparisons of preprocedural and postprocedural data were calculated by paired samples Wilcoxon test.

For analysis of collinearity, Pearson correlation coefficients were calculated.

Receiver operating characteristic curves and their corresponding areas under the curve (AUCs) were calculated to evaluate the performance of various RV-PA coupling indices in predicting 1-year mortality after TAVR. The DeLong test was employed to assess significant differences in the predictive accuracy between these indices, as reflected by their AUCs. Furthermore, receiver operating characteristic curves were utilized to determine optimal thresholds (based on the Youden index) of RV-PA coupling indices for predicting 1-year all-cause mortality after TAVR.

To evaluate the improvement in risk stratification provided by the RV-PA coupling concept compared to the traditional definition of PH, defined as mPAP levels ≥ 25 mmHg, we calculated the net reclassification index. Again, the primary outcome measure for this analysis was 1-year all-cause mortality following TAVR.

Survival was illustrated using the Kaplan–Meier method, and the log-rank test was applied to compare survival rates. Moreover, a Cox proportional hazards model was used to estimate hazard ratios.

A *p* value ≤0.05 was considered to indicate statistical significance.

All statistical analyses were performed using R statistical software (R version 3.6.3; R Foundation for Statistical Computing, Vienna, Austria).

## Results

### Three-Hundred Thirty-Three Patients With Complete Echocardiography and Right Heart Catheterization Before TAVR for Severe AS Constitute the Study Population

**Figure 1:**
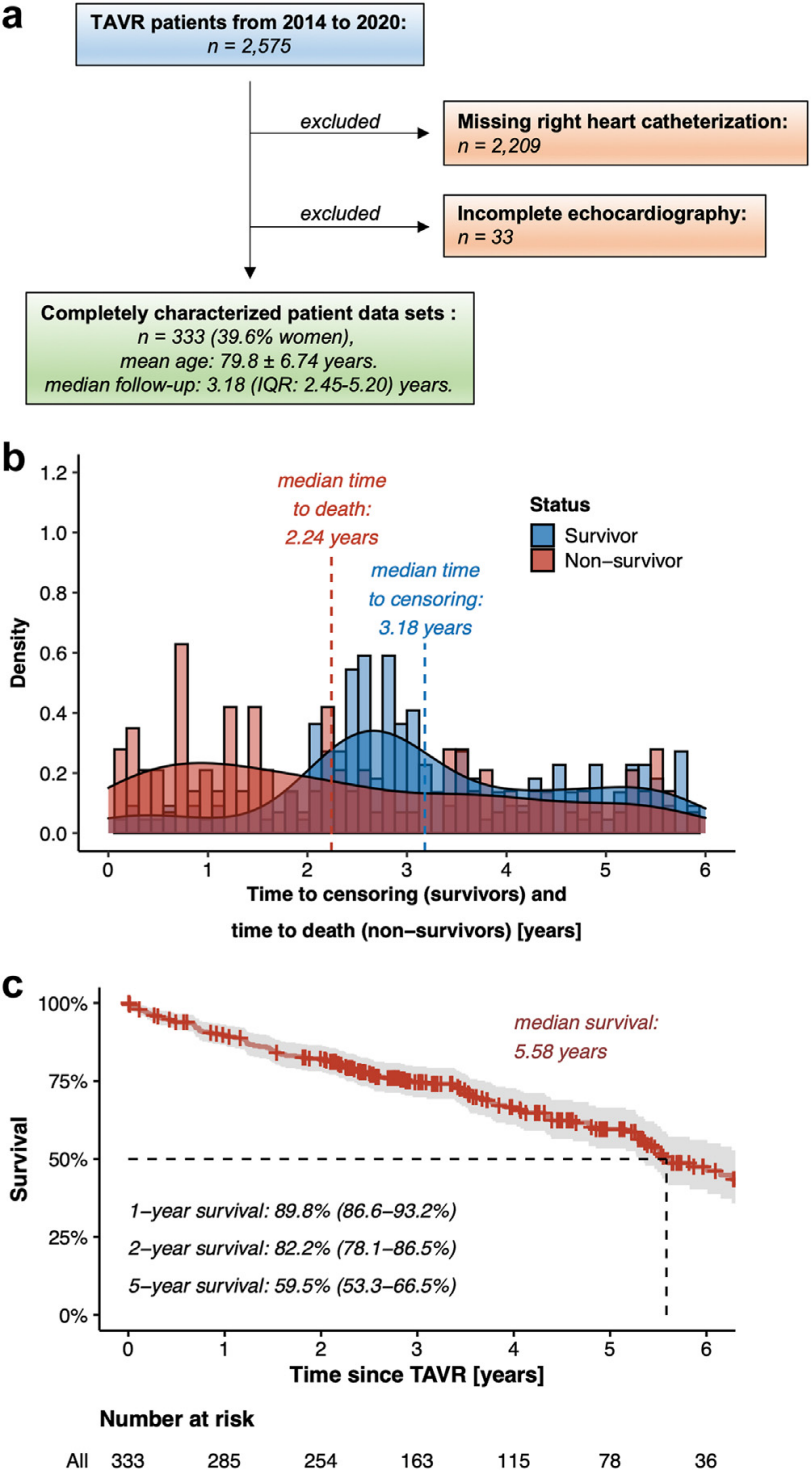
**General information about the study population from recruitment to follow-up.** (a) Flow chart for patient recruitment. (b) Density plot illustrating time to death and time to censoring for the entire study population. (c) Kaplan–Meier survival plot for the entire study population. Abbreviation: TAVR, transcatheter aortic valve replacement.

In total, this bicentric registry contained 2575 patients undergoing TAVR for severe AS between 2014 and 2020. At first, 2209 patients were excluded due to missing preprocedural right heart catheterization; secondly, TAPSE and/or sPAP assessment was absent in 33 patients, so that the detailed analysis was performed on a data set including 333 patients (Figure 1a). The mean age of the study population was 79.8 ± 6.74 years, and 132 out of 333 patients were female (39.6%) (Table 1). The mean AVA was 0.781 ± 0.207 cm^2^ (Table 2), and patients typically presented with dyspnea corresponding to New York Heart Association functional class III (54.1%) or IV (9.91%). Overall, 127 deaths among 333 enrolled patients were recorded, with 50% of deaths occurring within 2.24 years after TAVR (Figure 1b). Moreover, survivors were traced on a median follow-up time of 3.18 years (interquartile range: 2.45-5.20 years), resulting in a median survival of 5.58 years (Figure 1c).

**Table 1:** Demographic and clinical characteristics in accordance with RV-PA coupling

	All patients (*n* = 333)	RV-PA coupling (as TAPSE/sPAP_invasive_)		*p* value
		Preserved (*n* = 225)	Reduced (*n* = 108)	
Age, mean ± SD [95% CI], years	79.8 ± 6.74 [79.1-80.6]	79.8 ± 6.21 [79.0-80.6]	79.9 ± 7.77 [78.5-81.4]	0.334
Women, No. (%)	132 (39.6%)	95 (42.2%)	37 (34.3%)	0.204
BMI mean ± SD [95% CI], kg/m^2^	26.8 ± 4.47 [26.3-27.2]	26.9 ± 4.42 [26.3-27.5]	26.4 ± 4.58 [25.6-27.3]	0.410
Arterial hypertension, No. (%)	306 (92.2%)	202 (89.8%)	104 (96.3%)	0.084
Diabetes mellitus, No. (%)	98 (29.5%)	66 (29.3%)	32 (29.6%)	1.00
NYHA functional class III	180 (54.1%)	113 (50.2%)	67 (62.0%)	0.056
NYHA functional class IV	33 (9.91%)	15 (6.67%)	18 (16.7%)	0.008
EuroSCORE, mean ± SD [95% CI], %	18.2 ± 14.6 [16.6-19.8]	14.4 ± 10.3 [13.0-15.8]	26.1 ± 18.6 [22.6-29.7]	<0.0001
eGFR, mean ± SD [95% CI], mL/min	59.6 ± 20.8 [57.4-61.9]	62.6 ± 19.6 [60.0-65.2]	53.4 ± 21.9 [49.3-57.6]	<0.0001
CAD, No. (%)	282 (84.9%)	192 (85.3%)	90 (83.3%)	0.686
COPD, No. (%)	45 (13.6%)	20 (8.89%)	25 (23.2%)	0.001
Atrial fibrillation and/or flutter, No. (%)	148 (44.4%)	68 (30.2%)	80 (74.1%)	<0.0001

Abbreviations: BMI, body mass index; CAD, coronary artery disease; CI, confidence interval; COPD, chronic obstructive pulmonary disease; eGFR, estimated glomerular filtration rate; NYHA, New York Heart Association; RV-PA, right ventricular to pulmonary artery; sPAP, systolic pulmonary artery pressure; TAPSE, tricuspid annular plane systolic excursion.

**Table 2:** Comparison of preprocedural echocardiographic and hemodynamic characteristics in accordance with RV-PA coupling

	All patients (*n* = 333)	RV-PA coupling (as TAPSE/sPAP_invasive_)		*p* value
		Preserved (*n* = 225)	Reduced (*n* = 108)	
AVA, mean ± SD [95% CI], cm^2^	0.781 ± 0.207 [0.759-0.803]	0.785 ± 0.203 [0.758-0.812]	0.773 ± 0.214 [0.732-0.814]	0.716
AVG_mean_, mean ± SD [95% CI], mmHg	39.6 ± 15.7 [37.9-41.3]	41.7 ± 14.5 [39.8-43.6]	35.1 ± 17.2 [31.8-38.4]	<0.0001
Cardiac output, mean ± SD [95% CI], L/min	4.93 ± 1.22 [4.80-5.07]	5.12 ± 1.18 [4.96-5.27]	4.56 ± 1.20 [4.33-4.79]	<0.0001
LVEF, mean ± SD [95% CI], %	52.6 ± 11.4 [51.4-53.9]	55.8 ± 8.19 [54.8-56.9]	45.9 ± 14.0 [43.2-48.6]	<0.0001
LVEDD, mean ± SD [95% CI], mm	47.1 ± 8.63 [46.2-48.1]	45.3 ± 7.62 [44.2-46.3]	50.9 ± 9.34 [49.1-52.7]	<0.0001
sPAP (echocardiography), mean ± SD [95% CI], mmHg	47.4 ± 16.1 [45.4-49.3]	41.7 ± 13.4 [39.6-43.7]	57.9 ± 15.3 [54.7-61.0]	<0.0001
sPAP (invasive), mean ± SD [95% CI], mmHg	44.6 ± 16.7 [42.8-46.4]	36.4 ± 10.0 [35.1-37.8]	61.8 ± 14.7 [59.0-64.6]	<0.0001
dPAP (invasive), mean ± SD [95% CI], mmHg	17.5 ± 8.44 [16.6-18.4]	13.8 ± 5.86 [13.0-14.5]	25.2 ± 7.75 [23.7-26.7]	<0.0001
mPAP (invasive), mean ± SD [95% CI], mmHg	28.7 ± 11.5 [27.4-29.9]	23.0 ± 7.32 [22.1-24.0]	40.4 ± 9.76 [38.5-42.3]	<0.0001
mPCWP (invasive), mean ± SD [95% CI], mmHg	17.7 ± 8.46 [16.8-18.6]	14.1 ± 6.40 [13.3-15.0]	25.2 ± 7.24 [23.8-26.6]	<0.0001
PVR, mean ± SD [95% CI], WU	2.40 ± 1.57 [2.23-2.57]	1.83 ± 0.932 [1.71-1.95]	3.59 ± 1.92 [3.22-3.96]	<0.0001
TAPSE, mean ± SD [95% CI], mm	19.7 ± 5.04 [19.1-20.2]	21.5 ± 4.37 [21.0-22.1]	15.8 ± 4.05 [15.0-16.5]	<0.0001
Right midventricular diameter, mean ± SD [95% CI], mm	29.6 ± 6.75 [28.9-30.4]	28.8 ± 6.51 [27.9-29.7]	31.3 ± 6.96 [29.9-32.7]	0.003
LA area, mean ± SD [95% CI], cm^2^	26.3 ± 8.35 [25.4-27.3]	24.7 ± 7.62 [23.7-25.7]	29.7 ± 8.80 [28.0-31.5]	<0.0001
RA area, mean ± SD [95% CI], cm^2^	20.6 ± 7.56 [19.8-21.5]	18.6 ± 6.27 [17.7-19.4]	24.8 ± 8.28 [23.1-26.4]	<0.0001
“Classical” low-flow, low-gradient AS	56 (16.9%)	19 (8.44%)	37 (34.3%)	<0.0001
LV dysfunction (LVEF < 50%)	92 (27.8)	35 (15.6%)	57 (52.8%)	<0.0001
PH (mPAP ≥ 25 mmHg)	185 (55.6%)	83 (36.9%)	102 (94.4%)	<0.0001
RV dysfunction (TAPSE ≤ 16 mm)	85 (25.5%)	25 (11.1%)	60 (55.6%)	<0.0001
MR ≥ III/IV°, No (%)	32 (9.61%)	8 (3.56%)	24 (22.2%)	<0.0001
TR ≥ III/IV°, No (%)	30 (9.01%)	13 (5.78%)	17 (15.7%)	0.006

Abbreviations: AVA, aortic valve area; AVG_mean_, mean aortic valve gradient; CI, confidence interval; dPAP, diastolic pulmonary artery pressure; LA area, left atrial area; LV dysfunction, left ventricular dysfunction; LVEDD, left ventricular end-diastolic diameter; LVEF, left ventricular ejection fraction; mPAP, mean pulmonary artery pressure; mPCWP, mean postcapillary wedge pressure; MR, mitral regurgitation; PH, pulmonary hypertension; PVR, pulmonary vascular resistance; RA area, right atrial area; RV dysfunction, right ventricular dysfunction; RV-PA, right ventricular to pulmonary artery; sPAP, systolic pulmonary artery pressure; TAPSE, tricuspid annular plane systolic excursion; TR, tricuspid regurgitation.

### Invasive Assessment of sPAP Levels Refines the Predictive Value of TAPSE/sPAP Ratio to Foresee 1-Year All-Cause Mortality After TAVR

**Figure 2:**
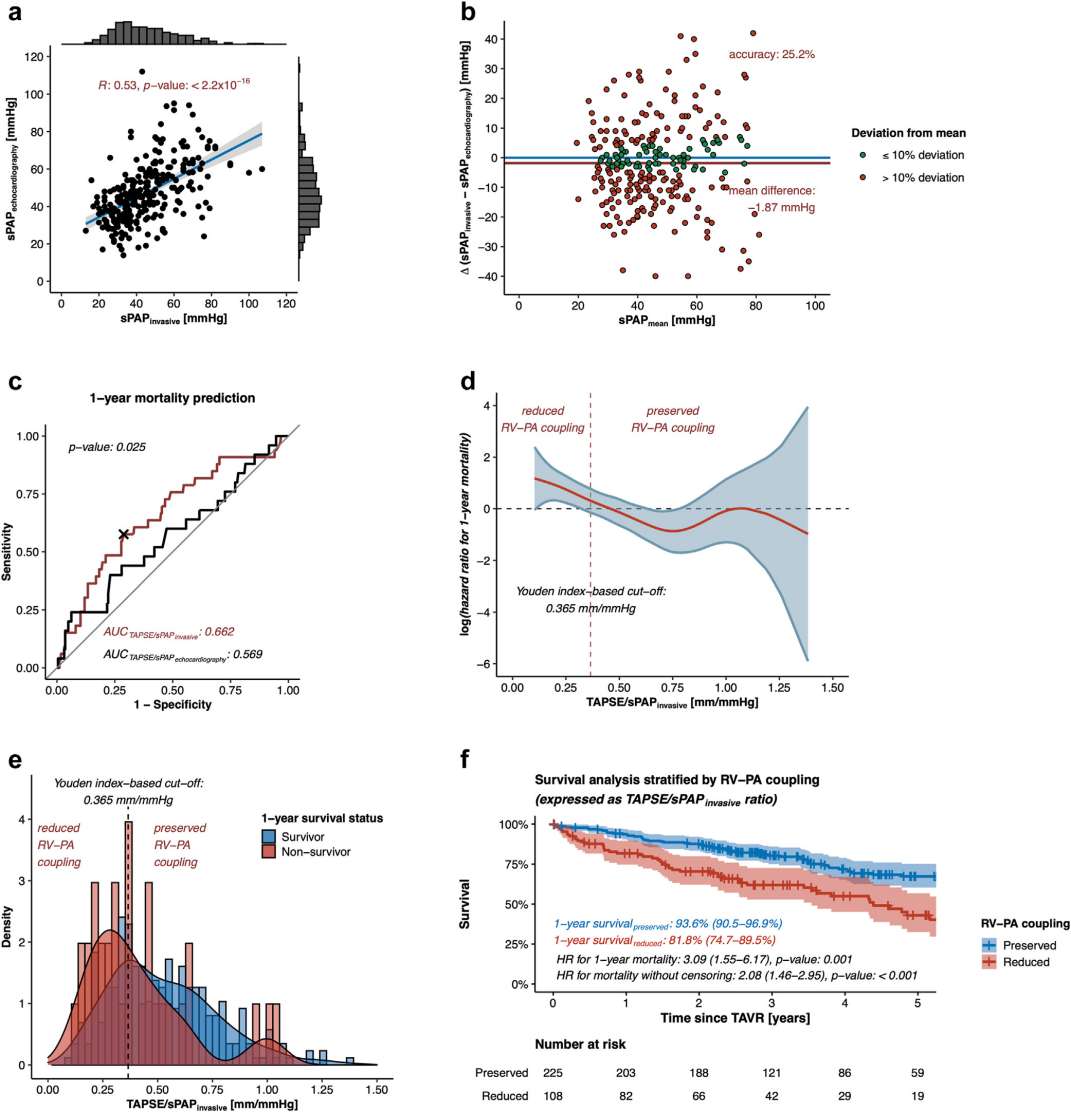
**Testing the superiority of TAPSE/sPAP**_**invasive**_**over TAPSE/sPAP**_**echocardiography**_**in predicting 1-year mortality in patients with severe AS undergoing TAVR.** (a) Linear regression plot assessing the correlation (*R* = correlation coefficient by Pearson) between invasive and echocardiographic sPAP assessments. Blue line: regression line. Gray area: 95% confidence interval. (b) Bland Altman plot assessing accuracy as a relative deviation from the respective mean value of invasive and echocardiographic sPAP assessments. Accuracy was defined as the proportion of invasive and echocardiographic sPAP assessments with ≤ 10% deviation from the respective mean value. Horizontal red line: mean difference between invasive and echocardiographic sPAP assessments. (c) Receiver operating characteristic curves comparing the performance of RV-PA coupling models to predict mortality after TAVR. The black cross indicates the location of the Youden index-based threshold of TAPSE/sPAP_invasive_ level to best predict 1-year mortality after TAVR. (d) Spline plot demonstrating the hazard ratio for 1-year mortality after TAVR per TAPSE/sPAP_invasive_ level. (e) Density plot illustrating the dichotomization of patients in accordance with reduced or preserved RV-PA coupling. (f) Kaplan–Meier survival plot in accordance with RV-PA coupling. Abbreviations: AS, aortic stenosis; RV-PA, right ventricular to pulmonary artery; sPAP, systolic pulmonary artery pressure; TAPSE, tricuspid annular plane systolic excursion; TAVR, transcatheter aortic valve replacement.

**Figure 3:**
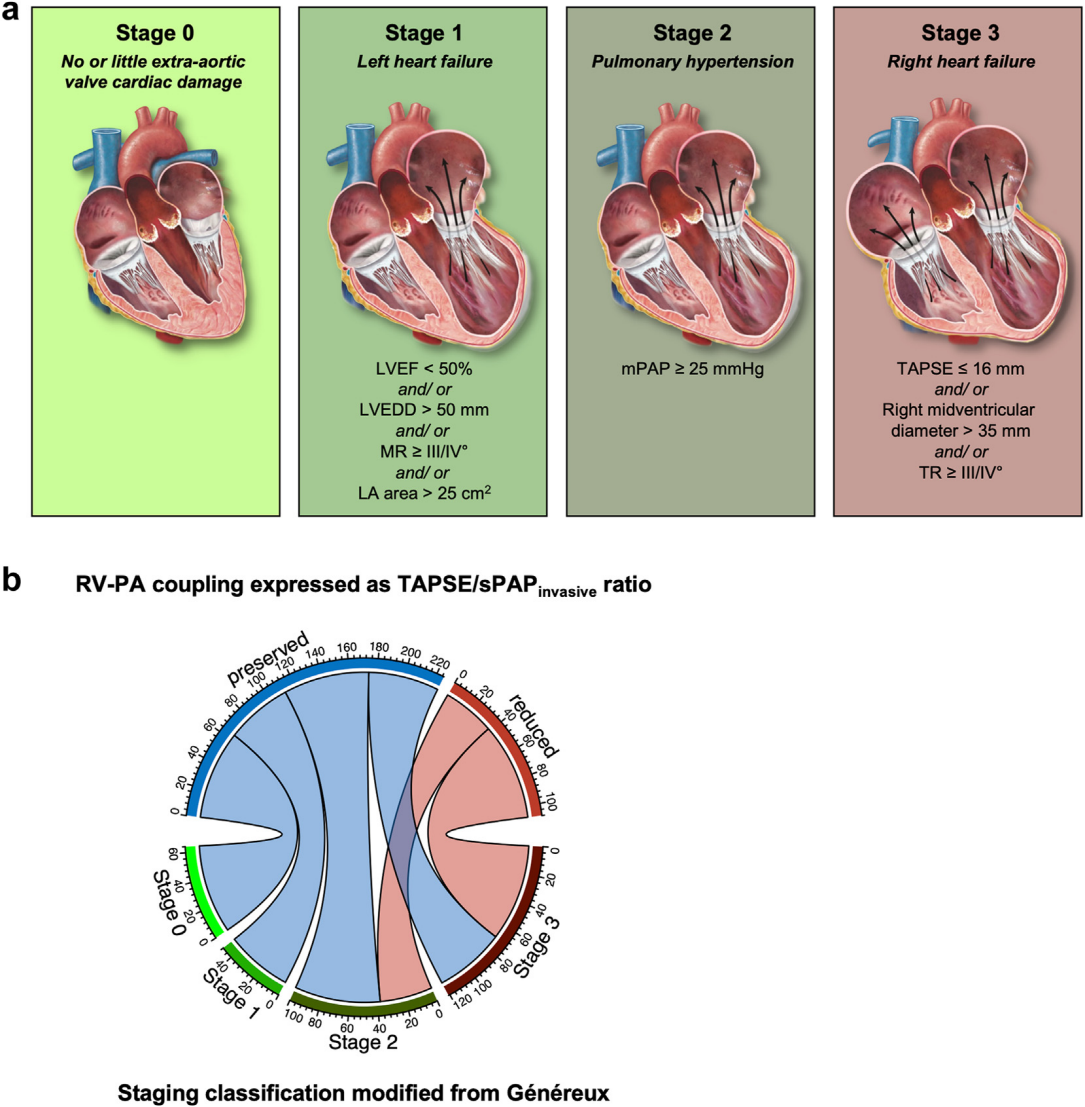
**Relationship between RV-PA coupling status and extra-aortic valve cardiac damage as assessed by a modified staging classification from Généreux.**^8^ (a) Sequential staging classification modified from Généreux based on the extent of extra-aortic valvular cardiac damage. (b) Chord diagram to relate RV-PA coupling status and extra-aortic valve cardiac damage. Abbreviations: LA area, left atrial area; LVEDD, left ventricular end-diastolic diameter; LVEF, left ventricular ejection fraction; mPAP, mean pulmonary artery pressure; MR, mitral regurgitation; RV-PA, right ventricular to pulmonary artery; sPAP, systolic pulmonary artery pressure; TAPSE, tricuspid annular plane systolic excursion; TR, tricuspid regurgitation.

**Supplemental Figure 1:**
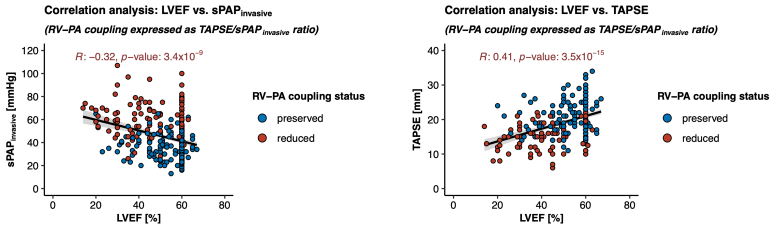


Assessment of sPAP levels by echocardiography and right heart catheterization showed only moderate, yet highly significant correlation (Pearson correlation coefficient *R*: 0.53, *p* value: <0.0001) (Figure 2a). The mean difference between the methods was -1.87 mmHg (95% CI: -3.74 to 0.11 mmHg) (Figure 2b). Relating sPAP levels as assessed by echocardiography and right heart catheterization to TAPSE levels to calculate TAPSE/sPAP_echocardiography_ and TAPSE/sPAP_invasive_ finally revealed that TAPSE/sPAP_invasive_ is superior to TAPSE/sPAP_echocardiography_ in predicting 1-year all-cause mortality after TAVR (AUC: 0.662 [95% CI: 0.559-0.764] vs. 0.569 [95% CI: 0.440-0.697], respectively, *p* value: 0.025) (Figure 2c). Calculating the Youden index to dichotomize the study population according to TAPSE/sPAP_invasive_ ratio resulted in an ideal threshold of 0.365 mm/mmHg with respect to 1-year all-cause mortality following TAVR; reduced RV-PA coupling is hence defined by low TAPSE/sPAP_invasive_ levels (<0.365 mm/mmHg), while preserved RV-PA coupling is defined by high TAPSE/sPAP_invasive_ levels (≥ 0.365 mm/mmHg) (Figure 2d-e). Kaplan–Meier analysis revealed that patients with reduced RV-PA coupling feature a significantly lower survival after TAVR in comparison to patients with preserved RV-PA coupling (1-year survival: 81.8% [95% CI: 74.7%-89.5%] vs. 93.6% [95% CI: 90.5%-96.9%], hazard ratio for 1-year mortality: 3.09 [95% CI: 1.55-6.17], *p* value: 0.001) (Figure 2f). Our analysis of the net reclassification index revealed that applying the RV-PA coupling concept led to a net reclassification improvement of 0.172, which was statistically significant with a *p* value of 0.045. This improvement indicates a positive shift in risk stratification for 1-year all-cause mortality following TAVR. Specifically, the RV-PA coupling categorization correctly identified a higher risk in a significant proportion of patients who died within 1 ​year after TAVR, while minimizing the misclassification of survivors into the higher risk category. This finding underscores the enhanced prognostic value of the RV-PA coupling metric over the traditional PH definition solely based on mPAP levels. Whilst AVA was similar between patients with reduced and preserved RV-PA coupling (0.773 ± 0.214 cm^2^ vs. 0.785 ± 0.203 cm^2^, *p* value: 0.716) (Table 2), patients with reduced RV-PA coupling were more often diagnosed with chronic obstructive pulmonary disease (COPD) (23.2 vs. 8.89%, *p* value: 0.001) and with atrial fibrillation (74.1 vs. 30.2%, *p* value: <0.0001), and subsequently suffered from more severe dyspnea (patients presenting with New York Heart Association functional class IV: 16.7 vs. 6.67%, *p* value: 0.008) (Table 1). Patients with reduced RV-PA coupling presented with reduced left ventricular ejection fraction (45.9% ± 14.0% vs. 55.8% ± 8.19%, *p* value: <0.0001), enlargement of left ventricular end-diastolic diameter (50.9 ± 9.34 mm vs. 45.3 ± 7.62 mm, *p* value: <0.0001) and left atrial area (29.7 ± 8.80 cm^2^ vs. 24.7 ± 7.62 cm^2^, *p* value: <0.0001), and with elevated mean postcapillary wedge pressure levels (25.2 ± 7.24 mmHg vs. 14.1 ± 6.40 mmHg, *p* value: <0.0001). Moreover, patients with reduced RV-PA coupling showed higher sPAP levels as assessed by right heart catheterization (61.8 ± 14.7 mmHg vs. 36.4 ± 10.0 mmHg, *p* value: <0.0001), and lower TAPSE levels (15.8 ± 4.05 mm vs. 21.5 ± 4.37 mm, *p* value: <0.0001). Correlation analyses verified the inverse relationship between left ventricular ejection fraction (LVEF) and sPAP_invasive_ levels (Pearson correlation coefficient *R*: -0.32, *p* value: <0.0001), and concurrently, the positive relationship between LVEF and TAPSE levels (Pearson correlation coefficient *R*: 0.41, *p* value: <0.0001) (Supplemental Figure 1). Concomitant to the enlargement of the right ventricle (right midventricular diameter: 31.3 ± 6.96 mm vs. 28.8 ± 6.51 mm, *p* value: 0.003) and right atrium (right atrial area: 24.8 ± 8.28 cm^2^ vs. 18.6 ± 6.27 cm^2^, *p* value: <0.0001), patients with reduced RV-PA coupling were also more often diagnosed with severe TR (15.7 vs. 5.78%, *p* value: 0.006). Notably, a “classical” low-flow, low-gradient AS was more often detected in patients with reduced RV-PA coupling than in those with preserved RV-PA coupling (34.3 vs. 8.44%, *p* value: <0.0001) (Table 2), even though the prevalence of coronary artery disease was similar among those groups (83.3 vs. 85.3%, *p* value: 0.686) (Table 1). Furthermore, all patients with reduced RV-PA coupling were found to be in the advanced stages of extra-aortic valve cardiac damage, with PH present in 38 out of 108 patients (35.2%), and right heart failure observed in 70 out of 108 patients (64.8%) (Figure 3).

### Patients With Reduced RV-PA Coupling Feature Persistent Structural and Functional Damage of the Right Heart After TAVR

**Figure 4:**
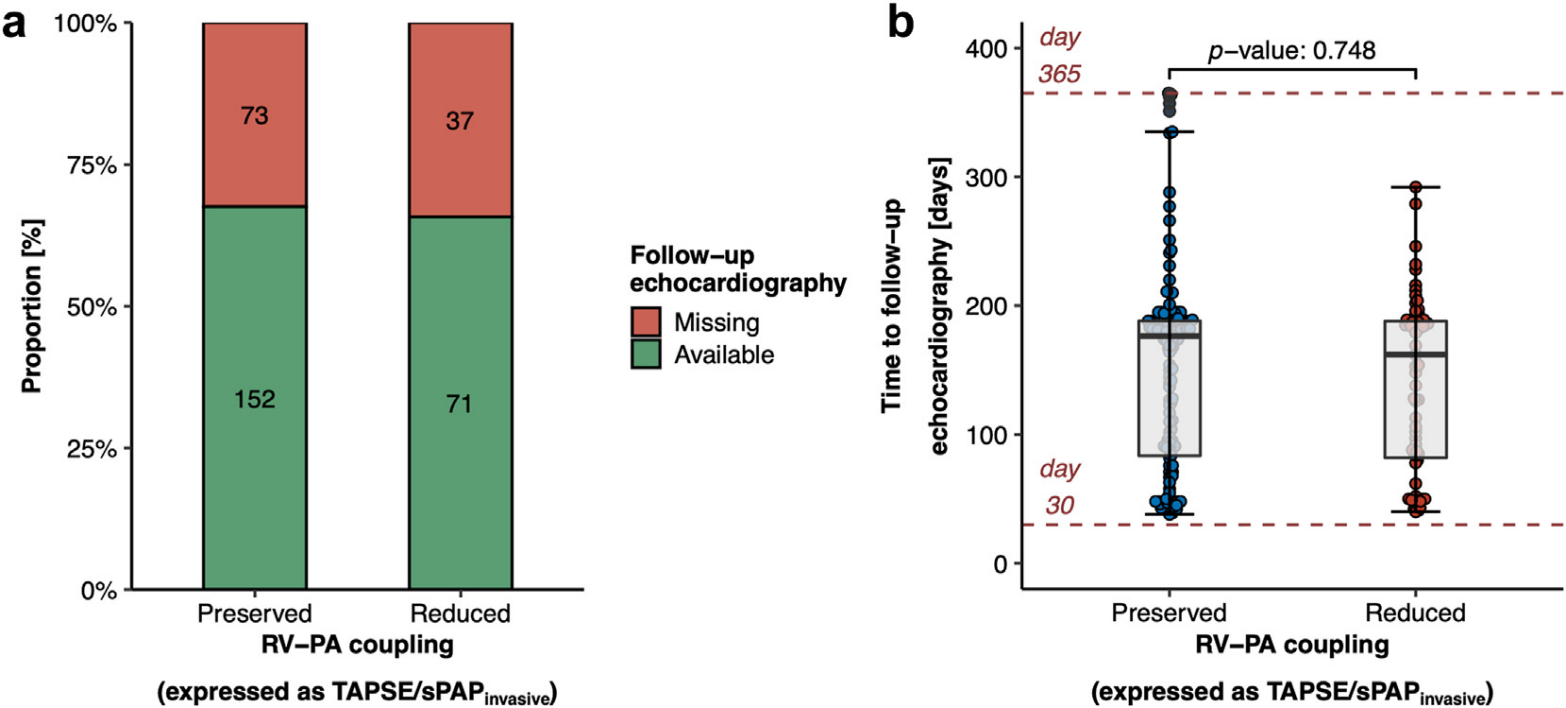
**Availability of follow-up echocardiography data.** (a) Proportion of patients with valid follow-up echocardiography. (b) Comparison of time from TAVR to follow-up echocardiography. Abbreviations: RV-PA, right ventricular to pulmonary artery; sPAP, systolic pulmonary artery pressure; TAPSE, tricuspid annular plane systolic excursion; TAVR, transcatheter aortic valve replacement.

**Figure 5:**
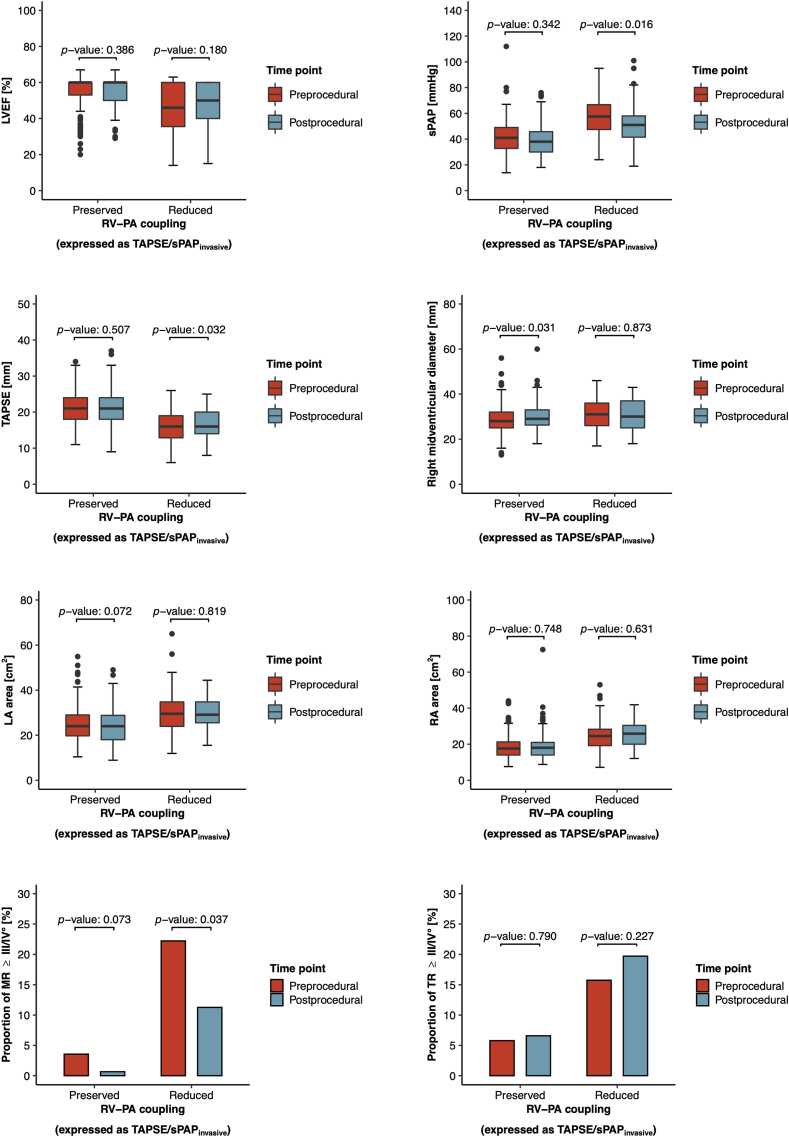
**Comparison of echocardiographic parameters before and after TAVR in accordance with RV-PA coupling**. Abbreviations: LA area, left atrial area; LVEF, left ventricular ejection fraction; MR, mitral regurgitation; RA area, right atrial area; RV-PA, right ventricular to pulmonary artery; sPAP, systolic pulmonary artery pressure; TAPSE, tricuspid annular plane systolic excursion; TAVR, transcatheter aortic valve replacement; TR, tricuspid regurgitation.

Among the initial patient cohort of 333 patients with complete echocardiography and right heart catheterization data obtained before TAVR, follow-up echocardiography was available for 223 patients (67.0%). Importantly, equal proportions of follow-up echocardiography were available for patients with preserved and reduced RV-PA coupling (Figure 4a), and also the time from TAVR to follow-up echocardiography was statistically indifferent (mean time from TAVR to follow-up echocardiography: 148 ± 74.0 [95% CI: 138-157] days; Figure 4b). Follow-up echocardiography revealed that the proportion of patients presenting with severe MR was significantly lower after TAVR than before TAVR (from 9.61% at baseline to 4.04% at follow-up, *p* value: 0.004). Moreover, the degree of backward transmission of left-sided filling pressures expressed as sPAP levels was significantly ameliorated after TAVR (from 47.4 ± 16.1 mmHg at baseline to 43.5 ± 15.2 mmHg at follow-up, *p* value: 0.015) (Table 3). This reduction in sPAP levels was mainly seen in patients with reduced RV-PA coupling (from 57.9 ± 15.3 mmHg at baseline to 52.3 ± 16.4 mmHg at follow-up, *p* value: 0.016) (Figure 5, Table 3). Furthermore, right ventricular function expressed as TAPSE slightly improved in patients with reduced RV-PA coupling upon TAVR (from 15.8 ± 4.05 mm at baseline to 16.6 ± 4.05 mm at follow-up, *p* value: 0.032). Yet, structural alterations of the right heart such as right ventricular and atrial dilatation showed no reverse remodeling in patients with reduced RV-PA coupling upon TAVR, nor could the prevalence of concomitant severe TR be reduced (from 15.7% at baseline to 19.7% at follow-up, *p* value: 0.227) (Figure 5). In comparison to patients with preserved RV-PA coupling, patients with reduced RV-PA coupling showed persistently higher pulmonary artery pressure levels (sPAP: 52.3 ± 16.4 mmHg vs. 39.0 ± 12.3 mmHg, *p* value: <0.0001), worse right ventricular systolic function (TAPSE: 16.6 ± 4.05 mm vs. 21.6 ± 4.81 mm, *p* value: <0.0001), and they were more often diagnosed with concomitant severe TR (19.7 vs. 6.58%, *p* value: 0.007) at echocardiographic follow-up (Table 3).

**Table 3:** Comparison of echocardiographic follow-up data in accordance with RV-PA coupling

	All patients (*n* = 223)	RV-PA coupling (as TAPSE/sPAP_invasive_)		*p* value
		Preserved (*n* = 152)	Reduced (*n* = 71)	
LVEF, mean ± SD [95% CI], %	53.2 ± 9.93 [51.9-54.5]	55.8 ± 6.95 [54.7-56.9]	47.5 ± 12.7 [44.5-50.5]	<0.0001
sPAP, mean ± SD [95% CI], mmHg	43.5 ± 15.2 [41.3-45.7]	39.0 ± 12.3 [36.8-41.2]	52.3 ± 16.4 [48.2-56.5]	<0.0001
Right midventricular diameter, mean ± SD [95% CI], mm	30.0 ± 6.08 [29.1-30.9]	29.7 ± 5.90 [28.6-30.8]	30.7 ± 6.44 [29.0-32.4]	0.284
TAPSE, mean ± SD [95% CI], mm	20.0 ± 5.13 [19.3-20.6]	21.6 ± 4.81 [20.8-22.4]	16.6 ± 4.05 [15.7-17.6]	<0.0001
LA area, mean ± SD [95% CI], cm^2^	26.2 ± 7.95 [25.1-27.3]	24.4 ± 7.90 [23.1-25.7]	30.1 ± 6.54 [28.6-31.7]	<0.0001
RA area, mean ± SD [95% CI], cm^2^	21.0 ± 7.97 [19.9-22.0]	18.7 ± 7.40 [17.4-19.9]	25.7 ± 6.98 [24.1-27.4]	<0.0001
MR ≥ III/IV°, No (%)	9 (4.04%)	1 (0.658%)	8 (11.3%)	0.001
TR ≥ III/IV°, No (%)	24 (10.8%)	10 (6.58%)	14 (19.7%)	0.007

Abbreviations: CI, confidence interval; LA area, left atrial area; LVEF, left ventricular ejection fraction; MR, mitral regurgitation; RA area, right atrial area; RV-PA, right ventricular to pulmonary artery; sPAP, systolic pulmonary artery pressure; TAPSE, tricuspid annular plane systolic excursion; TR, tricuspid regurgitation.

## Discussion

### RV-PA Coupling Integrates PH and Right Ventricular Function as Prognostically Important Determinants of Survival Following TAVR

Identifying patients at risk for increased mortality is a core element in the practice of medicine. Yet, clinicians should not only strive to accurately predict mortality, but they must also take responsibility to understand and to explain to the patient the underlying factors for this prediction; our risk stratification model in accordance with TAPSE/sPAP_invasive_ ratio has 3 key advantages: i) it provides both pathophysiologically and prognostically meaningful information to patients and clinicians, ii) it is easy to comprehend and it can be calculated at the bedside (no black box algorithm for mortality prediction), and iii) drivers of mortality (such as persistent severe TR) can be anticipated and treated in the future to further improve survival.

### Stratification According to TAPSE/sPAP_invasive_ Ratio Allows to Predict the Trajectory of Extra-Aortic Valve Cardiac Damage Following TAVR

Survival in patients with severe AS inevitably depends on the recovery of extra-aortic valve cardiac damage. However, PH and right heart dysfunction can persist in a substantial number of cases after TAVR; and decline of right ventricular function and/or worsening of TR despite correction of AS are associated with a poor prognosis.^22^^,^^23^ Notably, patients with severe AS typically present with multiple comorbidities, and a plethora of contributors to PH and right heart dysfunction, such as COPD, will persist despite correction of severe AS and hence limit the expected benefit of TAVR. Our data show that the prevalence of COPD was significantly higher in patients with reduced RV-PA coupling than in patients with preserved RV-PA coupling (23.2 vs. 8.89%, *p* value: 0.001), and subsequently the sPAP levels at echocardiographic follow-up were persistently elevated in patients with reduced RV-PA coupling compared to patients with preserved RV-PA coupling (52.3 ± 16.4 mmHg vs. 39.0 ± 12.3 mmHg, *p* value: <0.0001). Similarly, the proportion of patients with severe TR among patients with reduced RV-PA coupling remained unchanged before and after TAVR (15.7% before and 19.7% after TAVR, *p* value: 0.227), meaning that stratification according to TAPSE/sPAP_invasive_ ratio allows to predict *ex ante* the trajectory of extra-aortic valve cardiac damage following TAVR — this competence will enable clinicians to invite high-risk patients for closer monitoring during follow-up and/or to focus on more aggressive treatment of comorbidities.

### On the Options to Improve Survival in Patients With Reduced TAPSE/sPAP_invasive_ Ratio

Correction of AS by TAVR and anticipated improvement of left heart hemodynamics resulted in a significant reduction in the proportion of patients suffering from concurrent severe secondary MR among patients with reduced RV-PA coupling (22.2% before and 11.3% after TAVR, *p* value: 0.037). Considering the possibly self-sustaining vicious circle of atrial fibrillation (diagnosed in 74.1% at baseline), persistent right ventricular dysfunction (TAPSE at follow-up: 16.6 ± 4.05 mm), persistent severe TR (diagnosed in 19.7% at follow-up) and persistent right atrial enlargement (right atrial area at follow-up: 25.7 ± 6.98 cm^2^) as found in patients with reduced RV-PA coupling, future studies should investigate potentially survival-prolonging effects of:

1) sinus rhythm restoration (difficult to achieve in patients with severely enlarged atria);
2) guideline-directed medical heart failure therapy (considering that left heart failure-specific therapeutics are not necessarily effective in right heart failure, as adaption of the right ventricle to pressure and volume overload differs from the left ventricle on a molecular level,^24^ possibly reflecting a distinct embryological origin and hemodynamic physiology^25^); and
3) accompanying transcatheter treatment of TR (notably, initial data from the Trial to Evaluate Cardiovascular Outcomes in Patients Treated with the Tricuspid Valve Repair System Pivotal (TRILUMINATE Pivotal) as the first randomized controlled analysis show that transcatheter tricuspid valve intervention improves the quality of life in an otherwise highly symptomatic patient population; however, no differences in rates of all-cause death or hospitalization for heart failure were evident at 1 year after randomization^26^).

In our study, rather than showing improvement, we observed a numerical increase in the proportion of patients with severe TR among those with reduced RV-PA coupling following TAVR. This trend might be attributed to factors such as persistently high pulmonary artery pressure levels, often driven by COPD, and advanced right heart remodeling. The factors, which appeared unaffected or insufficiently improved by the TAVR procedure, may have contributed to the persistence or even worsening of TR. It is well established that severe TR in patients undergoing TAVR is associated with poor prognosis.^23^^,^^27^^,^^28^ A recent study identified 3.4% of patients with TAVR as potential candidates for transcatheter tricuspid valve intervention,^29^ but larger prospective studies are mandatory to address the question where TR causally drives mortality and is therefore a clinically meaningful target, and where it represents an epiphenomenon indicating long-standing PH and right ventricular decompensation.^6^^,^^30^ Aiming to fill this gap, Fortmeier *et al*^31^ have proposed a proportionality concept for patients with severe TR undergoing transcatheter tricuspid valve intervention by relating the extent of tricuspid valve insufficiency (expressed as tricuspid valve effective regurgitant orifice area) to the afterload burden imposed from the pulmonary circulation (expressed as mPAP levels). Authors showed that patients with a tricuspid valve effective regurgitant orifice area /mPAP ratio ≤1.25 mm^2^/mmHg (defining proportionate TR) featured significantly lower 2-year survival rates after transcatheter tricuspid valve intervention than patients with disproportionate TR (56.6 vs. 69.6%, *p* value: 0.005). Even more important, 1-year mortality rate in patients with proportionate TR (32%) was similar to that described in literature for conservatively treated patients (between 26 and 36%).^32^^,^^33^ Applying this proportionality framework to patients with persistent severe TR after TAVR could hence improve clinical decision-making by addressing the crucial question: “Is this case of TR a prognostically relevant interventional target or does it merely represent an indicator of worse prognosis in patients suffering from PH?”

### Limitations

To the best of our knowledge, this is the largest database dealing with right heart catheterization data in TAVR patients. However, apart from being a retrospective, observational, non-randomized register study with inherent weaknesses, 3 major limitations of our analysis merit consideration.

Most importantly, right heart catheterization is not routinely performed before TAVR, but is reserved for cases where the severity of AS is inconclusive.^7^ As a consequence, invasive measurements of sPAP levels were not available in 2209 out of 2575 patients, which therefore had to be excluded. This exclusion process might have introduced a selection bias towards patients with more complex or uncertain clinical presentations, potentially skewing our study population towards those with a higher likelihood of heart failure or PH. Nonetheless, the representative character of our small but in-depth characterized study population has been validated in previous works^9^^,^^11^^,^^34^; most importantly, the proportion of patients with no or isolated postcapillary PH was found to be similar to that from other published datasets.^5^^,^^35^^,^^36^ It further remains elusive whether the gain in prognostic accuracy using TAPSE/sPAP_invasive_ rather than TAPSE/sPAP_echocardiography_ justifies right heart catheterization in all patients before TAVR, as right heart catheterization represents an invasive, cumbersome, and potentially dangerous procedure. Moreover, before influencing individual heart team decisions, the proposed cut-off value for TAPSE/sPAP_invasive_ ratio requires external validation.

Secondly, this study is based on data that were generated during clinical routine in a real-life scenario, meaning that no central core laboratory was involved to prevent any potential interobserver biases regarding echocardiography or right heart catheterization. It further needs to be acknowledged that pulmonary artery pressure levels may vary during a time course; and circumstances when pulmonary artery pressure levels were assessed by echocardiography may not have been the same as for right heart catheterization. Furthermore, right ventricular function was assessed by echocardiography alone. However, TAPSE as a parameter for right ventricular systolic function measures wall motion only at the basal level, which is particularly problematic in pathological remodeling.^37^ Moreover, some variables such as history of coronary artery disease were only captured as categorical variables in our study. It is plausible that the severity of coronary artery disease could have been more pronounced in patients with reduced RV-PA coupling. This increased severity might contribute to a decline in left ventricular systolic function and an enlargement of left ventricular diameters. Consequently, this could lead to a higher proportion of patients being diagnosed with low-flow, low-gradient AS in the group with reduced RV-PA coupling. While our analysis did not directly assess the severity of coronary artery disease, this factor should be considered when interpreting our findings, especially in the context of the observed relationship between reduced RV-PA coupling and low-flow, low-gradient AS. Furthermore, our study demonstrated a significant correlation, though not causation, between lower LVEF levels and elevated sPAP_invasive_ as well as decreased TAPSE levels as typically found in patients with reduced RV-PA coupling (illustrated in Supplemental Figure 1).

We acknowledge as a third major limitation that follow-up echocardiography was available for only 67.0% of patients. We can only speculate on the reasons for missing follow-up echocardiography, such as the rural structure in Bavaria, Germany, where patients would need to travel long distances (sometimes more than 200 km from the referral area) to reach one of our study centers; these long distances are particularly problematic in elderly, immobile patients. Moreover, some patients might have died between TAVR and follow-up echocardiography, even though the general survival rate of 89.8% at 1 year after TAVR (Figure 1c) indicates that this explanation is applicable only to a minority of patients. Most importantly, we have no selection bias in our data, as 1) equal proportions of patients were examined by follow-up echocardiography and 2) the time between TAVR and follow-up echocardiography was also equally long among patients with reduced and preserved RV-PA coupling.

## Conclusion

Three main conclusions can be drawn from this analysis Graphical Abstract:

1) TAPSE/sPAP_invasive_ is superior to TAPSE/sPAP_echocardiography_ in predicting 1-year mortality after TAVR.
2) Patients with reduced RV-PA coupling expressed as TAPSE/sPAP_invasive_ < 0.365 mm/mmHg show higher 1-year mortality rates than patients with preserved RV-PA coupling expressed as TAPSE/sPAP_invasive_ ≥ 0.365 mm/mmHg.
3) Even though TAVR ameliorates the pressure burden imposed from the pulmonary circulation, patients with initially reduced RV-PA coupling feature persistent right ventricular dysfunction and a high prevalence of concomitant severe TR — possibly indicating the next interventional target to further improve survival in this high-risk cohort.

Taken together — whenever available — TAPSE/sPAP_invasive_ ratio should be calculated to improve prognostication, guide future timing of intervention and/or to optimize the treatment of comorbidities in patients with severe AS undergoing TAVR.

## Ethics Statement

Planned and conducted in conformity with the Declaration of Helsinki, this study was approved by the local ethics committee, and all patients provided written informed consent for participation.

## Funding

Mark Lachmann received funding from the 10.13039/501100005713Technical University of Munich (Clinician Scientist Grant) and from the Else Kröner-Fresenius Foundation (Clinician Scientist Grant). Amelie Hesse received funding from the 10.13039/501100010578German Cardiac Society (DGK; Otto Hess Doctoral Scholarship).

## Data Sharing Statement

The data underlying this article will be shared on reasonable request to the corresponding author. All requests for raw and analyzed data and related materials will be reviewed by the Ethics Committee at Technical University of Munich, Germany. Any data and materials that can be shared will be released via a Material Transfer Agreement.

## Disclosure Statement

The authors report no conflict of interest.

## References

[1] Osnabrugge R.L.J., Mylotte D., Head S.J. (2013). Aortic stenosis in the elderly. J Am Coll Cardiol.

[2] Eveborn G.W., Schirmer H., Heggelund G., Lunde P., Rasmussen K. (2013). The evolving epidemiology of valvular aortic stenosis. The Tromsø Study. Heart.

[3] d’Arcy J.L., Coffey S., Loudon M.A. (2016). Large-scale community echocardiographic screening reveals a major burden of undiagnosed valvular heart disease in older people: the OxVALVE Population Cohort Study. Eur Heart J.

[4] Carabello B.A. (2013). Introduction to aortic stenosis. Circ Res.

[5] Weber L., Rickli H., Haager P.K. (2019). Haemodynamic mechanisms and long-term prognostic impact of pulmonary hypertension in patients with severe aortic stenosis undergoing valve replacement: impact of PH in severe aortic stenosis. Eur J Heart Fail.

[6] Cavalcante J.L., Simon M.A., Chan S.Y. (2017). Comprehensive right-sided assessment for transcatheter aortic valve replacement risk stratification: time for a change. J Am Soc Echocardiogr.

[7] Vahanian A., Beyersdorf F., Praz F. (2022). 2021 ESC/EACTS Guidelines for the management of valvular heart disease. Eur Heart J.

[8] Généreux P., Pibarot P., Redfors B. (2017). Staging classification of aortic stenosis based on the extent of cardiac damage. Eur Heart J.

[9] Lachmann M., Rippen E., Schuster T. (2021). Subphenotyping of patients with aortic stenosis by unsupervised agglomerative clustering of echocardiographic and hemodynamic data. JACC Cardiovasc Interv.

[10] Généreux P., Pibarot P., Redfors B. (2022). Evolution and prognostic impact of cardiac damage after aortic valve replacement. J Am Coll Cardiol.

[11] Lachmann M., Rippen E., Schuster T. (2022). Artificial intelligence-enabled phenotyping of patients with severe aortic stenosis: on the recovery of extra-aortic valve cardiac damage after transcatheter aortic valve replacement. Open Heart.

[12] Cahill T.J., Pibarot P., Yu X. (2022). Impact of right ventricle-pulmonary artery coupling on clinical outcomes in the PARTNER 3 trial. JACC Cardiovasc Interv.

[13] Karam N., Stolz L., Orban M. (2021). Impact of right ventricular dysfunction on outcomes after transcatheter edge-to-edge repair for secondary mitral regurgitation. JACC Cardiovasc Imaging.

[14] Brener M.I., Lurz P., Hausleiter J. (2022). Right ventricular-pulmonary arterial coupling and afterload reserve in patients undergoing transcatheter tricuspid valve repair. J Am Coll Cardiol.

[15] Lurz P., Orban M., Besler C. (2020). Clinical characteristics, diagnosis, and risk stratification of pulmonary hypertension in severe tricuspid regurgitation and implications for transcatheter tricuspid valve repair. Eur Heart J.

[16] Fortmeier V., Lachmann M., Körber M.I. (2022). Solving the pulmonary hypertension paradox in patients with severe tricuspid regurgitation by employing artificial intelligence. JACC Cardiovasc Interv.

[17] Humbert M., Kovacs G., Hoeper M.M. (2022). 2022 ESC/ERS Guidelines for the diagnosis and treatment of pulmonary hypertension. Eur Heart J.

[18] Sugiura A., Tanaka T., Kavsur R. (2023). Refining accuracy of RV–PA coupling in patients undergoing transcatheter tricuspid valve treatment. Clin Res Cardiol.

[19] Clavel M.-A., Magne J., Pibarot P. (2016). Low-gradient aortic stenosis. Eur Heart J.

[20] Otto C.M., Nishimura R.A., Bonow R.O. (2021). 2020 ACC/AHA guideline for the management of patients with valvular heart disease. J Am Coll Cardiol.

[21] Stekhoven D.J., Buhlmann P. (2012). MissForest--non-parametric missing value imputation for mixed-type data. Bioinformatics.

[22] Poch F., Thalmann R., Olbrich I. (2021). Changes of right ventricular function after transcatheter aortic valve replacement and association with outcomes. J Card Fail.

[23] Cremer P.C., Wang T.K.M., Rodriguez L.L. (2021). Incidence and clinical significance of worsening tricuspid regurgitation following surgical or transcatheter aortic valve replacement: analysis from the PARTNER IIA trial. Circ Cardiovasc Interv.

[24] Reddy S., Bernstein D. (2015). Molecular mechanisms of right ventricular failure. Circulation.

[25] Brade T., Pane L.S., Moretti A., Chien K.R., Laugwitz K.-L. (2013). Embryonic heart progenitors and cardiogenesis. Cold Spring Harb Perspect Med.

[26] Sorajja P., Whisenant B., Hamid N. (2023). Transcatheter repair for patients with tricuspid regurgitation. N Engl J Med.

[27] Lindman B.R., Maniar H.S., Jaber W.A. (2015). Effect of tricuspid regurgitation and the right heart on survival after transcatheter aortic valve replacement: insights from the placement of aortic transcatheter valves II inoperable cohort. Circ Cardiovasc Interv.

[28] Worku B., Valovska M.-T., Elmously A. (2018). Predictors of persistent tricuspid regurgitation after transcatheter aortic valve replacement in patients with baseline tricuspid regurgitation. Innovations (Phila).

[29] Tomii D., Okuno T., Praz F. (2021). Potential candidates for transcatheter tricuspid valve intervention after transcatheter aortic valve replacement. JACC Cardiovasc Interv.

[30] Nickenig G., Vogelhuber J. (2020). The tricuspid tragedy: from Cinderella to celebrity. Eur Heart J.

[31] Fortmeier V., Lachmann M., Unterhuber M. (2023). Epiphenomenon or prognostically relevant interventional target? A novel proportionality framework for severe tricuspid regurgitation. J Am Heart Assoc.

[32] Taramasso M., Benfari G., van der Bijl P. (2019). Transcatheter versus medical treatment of patients with symptomatic severe tricuspid regurgitation. J Am Coll Cardiol.

[33] Schlotter F., Miura M., Kresoja K.-P. (2021). Outcomes of transcatheter tricuspid valve intervention by right ventricular function: a multicentre propensity-matched analysis. EuroIntervention.

[34] Lachmann M., Rippen E., Rueckert D. (2022). Harnessing feature extraction capacities from a pre-trained convolutional neural network (VGG-16) for the unsupervised distinction of aortic outflow velocity profiles in patients with severe aortic stenosis. Eur Heart J Digit Health.

[35] O’Sullivan C.J., Wenaweser P., Ceylan O. (2015). Effect of pulmonary hypertension hemodynamic presentation on clinical outcomes in patients with severe symptomatic aortic valve stenosis undergoing transcatheter aortic valve implantation: insights from the New proposed pulmonary hypertension classification. Circ Cardiovasc Interv.

[36] Schewel J., Schmidt T., Kuck K.-H., Frerker C., Schewel D. (2019). Impact of pulmonary hypertension hemodynamic status on long-term outcome after transcatheter aortic valve replacement. JACC Cardiovasc Interv.

[37] Focardi M., Cameli M., Carbone S.F. (2015). Traditional and innovative echocardiographic parameters for the analysis of right ventricular performance in comparison with cardiac magnetic resonance. Eur Heart J Cardiovasc Imaging.

